# Wasserstein Uncertainty Estimation for Adversarial Domain Matching

**DOI:** 10.3389/fdata.2022.878716

**Published:** 2022-05-10

**Authors:** Rui Wang, Ruiyi Zhang, Ricardo Henao

**Affiliations:** ^1^Department of Electrical and Computer Engineering, Duke University, Durham, NC, United States; ^2^Department of Computer Science, Duke University, Durham, NC, United States

**Keywords:** Wasserstein, domain adaptation, uncertain, optimal transport, image classification

## Abstract

Domain adaptation aims at reducing the domain shift between a labeled source domain and an unlabeled target domain, so that the source model can be generalized to target domains without fine tuning. In this paper, we propose to evaluate the cross-domain transferability between source and target samples by domain prediction uncertainty, which is quantified via Wasserstein gradient flows. Further, we exploit it for reweighting the training samples to alleviate the issue of domain shift. The proposed mechanism provides a meaningful curriculum for cross-domain transfer and adaptively rules out samples that contain too much domain specific information during domain adaptation. Experiments on several benchmark datasets demonstrate that our reweighting mechanism can achieve improved results in both balanced and partial domain adaptation.

## 1. Introduction

Unsupervised domain adaptation transfers knowledge from a labeled source domain to an unlabeled target domain. The goal is to learn a shared latent representation of source and target samples, complemented with a classifier for accurate classification using the latent representation as input. During learning, the differences between source and target representations are minimized at a population (distribution) level, while the discriminative ability of the classifier is maximized using only the labeled source data. Subsequently, the learned classifier and representation can be used to predict on target samples without the need of manual labeling effort. With the popularity of Generative Adversarial Nets (GANs) (Goodfellow et al., 2014), recent approaches generally match the source and target latent representations (features) via adversarial training, where a domain discriminator is used to classify the source and target features while the feature encoders are trained adversarially so the the discriminator cannot tell the differences between the two domains.

**Figure 1:**
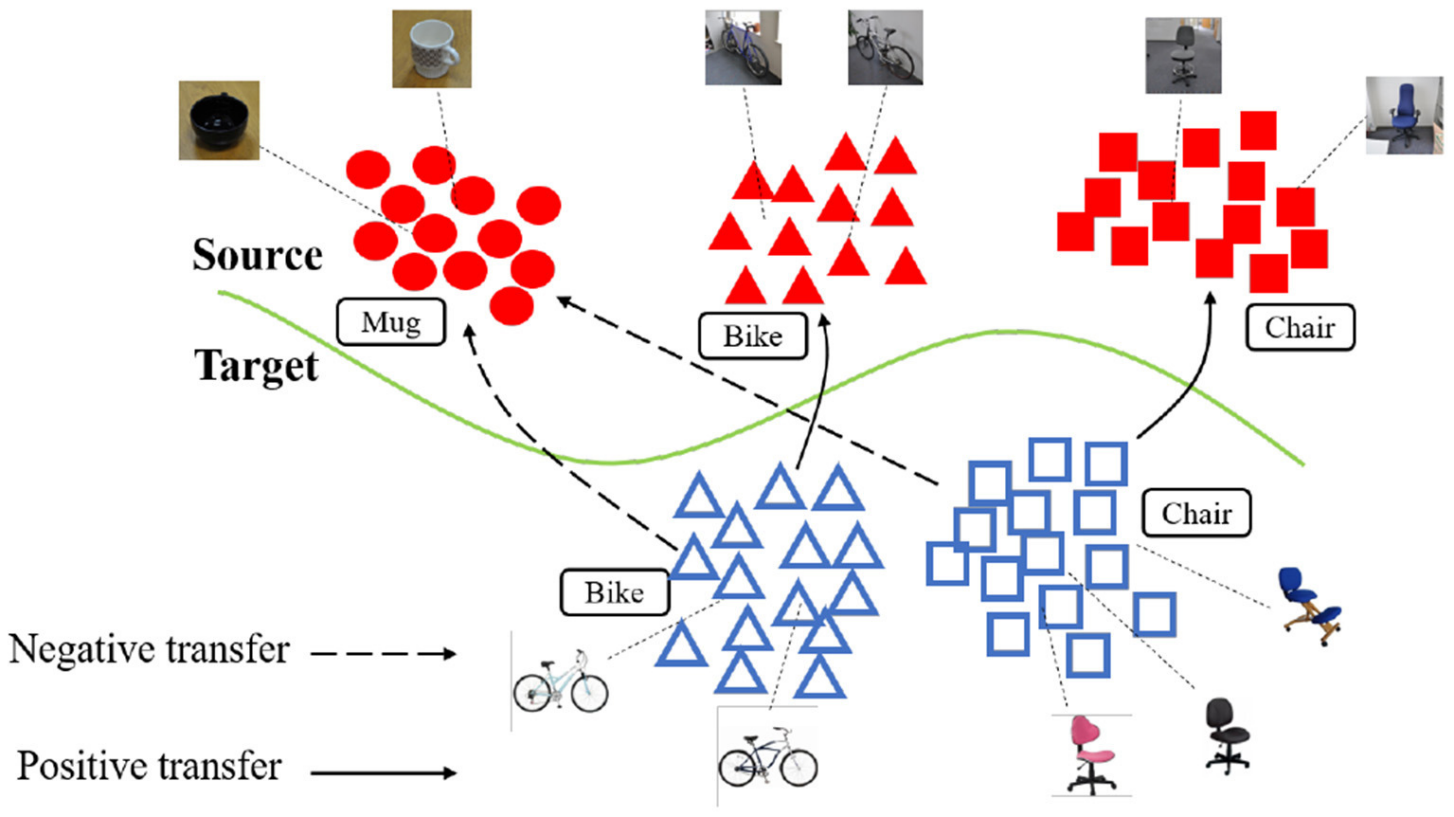
Negative transfer in partial domain adaptation. Source and target features are colored red and blue, respectively. Target instances will be negatively transferred to source classes that are absent in the target domain (e.g., “bike” to “mug”), resulting in poorly discriminative target features.

However, there are several problems with the current adversarial domain matching approaches: (*i*) The datasets may include samples that contain too much domain specific information. Matching with such samples may cause unreliable gradient and deterioration during training (Wen et al., 2019). (*ii*) Most of these approaches assume that the source and target are generated from the same set of classes, i.e., the *balanced domain adaptation*. In many real applications, however, in order to for the source knowledge to cover the target but with no information on target labels, we may have to collected a much larger source dataset with classes that do not present in the target domain. Such problem is described as the *partial domain adaptation* (Cao et al., 2017, 2018). As illustrated in Figure 1, these scenarios are challenging because simply matching the source and target feature distributions is likely to result in *negative transfer*. This happens because distribution matching may force observations from the target to be placed nearby source observations whose label is not present in the target, thus negatively impacting the quality of the learnt target representation. As a result, the adapted model may be sometimes worse than that trained on the source, as the target representation is poorly discriminative after adaptation.

In this paper, we propose to alleviate these issues via reweighting the source and target samples by their domain prediction uncertainty, where the uncertainty is estimated by employing a probabilistic domain discriminator. The weights based on uncertainty estimation is a measure of transferability of the source and target instances. It provides an adaptive curriculum that enables domain adaptation to initially focuses on domain uncertain samples that are close to the domain classification boundary (easy to transfer), then move to domain specific samples (difficult to transfer). Such a strategy can improve the stability of the adversarial learning process (Wen et al., 2019). Furthermore, source classes that are not presented in target can be easily identified with low domain uncertainty in partial domain adaptation. These classes will be down weighted, allowing the domain matching to focus on more target-related and informative source samples. In order to accurately estimate the domain prediction uncertainty, we account for the uncertainty in model parameters, i.e., by leveraging a Bayesian neural network (BNN) as the domain discriminator. In such case, the exact posterior is intractable, therefore, we approximate the posterior following Wasserstein gradient flows (WGFs) and a numerical solution of WGFs is proposed. We further define our *Wasserstein uncertainty estimation* via the mean entropy and the variance of the posterior predictions. *Wasserstein uncertainty estimation* can be easily integrated into current methods with adversarial domain matching, enabling appropriate uncertaint reweighting. Experimental results show significant improvement, obtaining improved results on both balanced and partial domain adaptation benchmarks.

## 2. Background

### 2.1. Adversarial Domain Matching

Assume we have a labeled *source* dataset, $D_{s}≜\left(X_{s},Y_{s}\right)$, where *X*_*s*_ and *Y*_*s*_ represent source inputs and labels, respectively. The source label, *Y*_*s*_ ∈ Y_*s*_, can take one of *K*_*s*_ distinct labels with probability *P*(*Y*_*s*_). We seek to leverage information in the source and a set of (unlabeled) *target* inputs, *X*_*t*_, to develop a target label classification model without knowing the target label *Y*_*t*_. Similarly, *Y*_*t*_ ∈ Y_*t*_ can take one of *K*_*t*_ distinct labels with probability *P*(*Y*_*t*_). Here we not only consider the standard scenario, denoted as *balanced domain adaptation*, where Y_*s*_ = Y_*t*_ and *P*(*Y*_*s*_) = *P*(*Y*_*t*_), but also the *partial domain adaptation*, where Y_*t*_ ⊂ Y_*s*_, i.e., the target labels are a true subset of the source labels, so *K*_*t*_ < *K*_*s*_. This is a common scenario in practice, where we need to transfer from a large source dataset to a smaller target with fewer number of classes.

**Figure 2:**
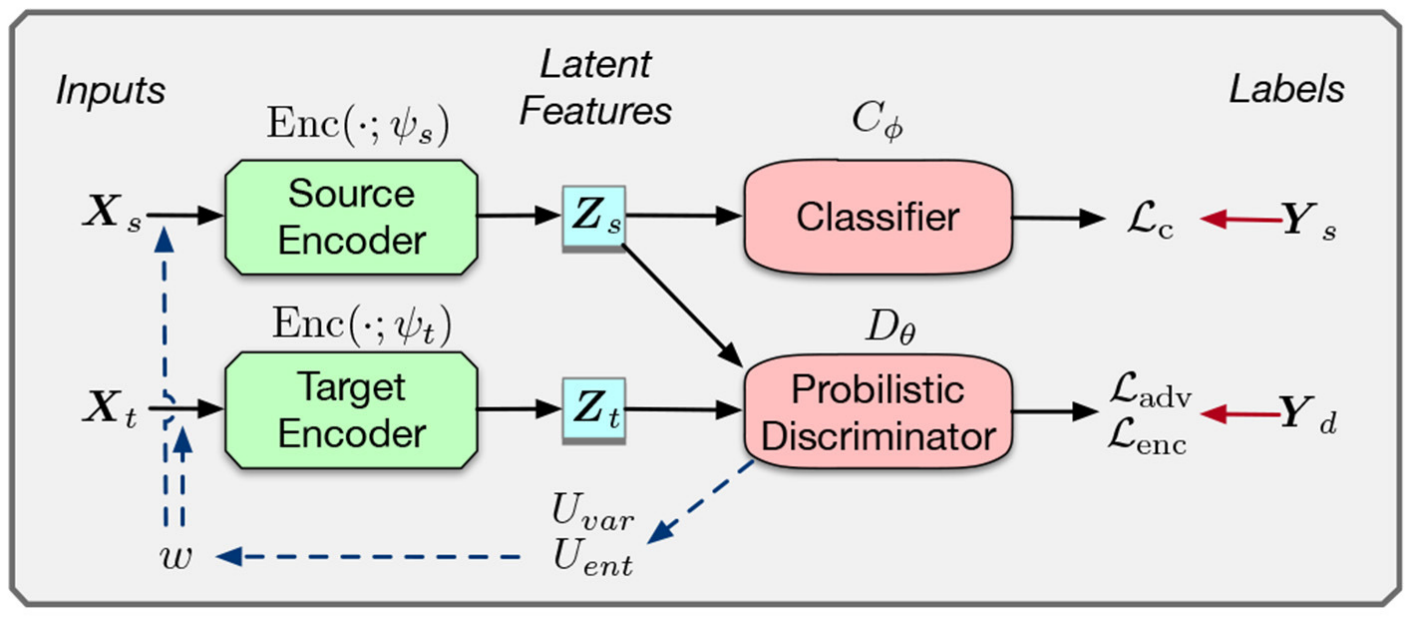
Framework for adversarial domain matching with uncertainty reweighting. *Y*_*s*_ and *Y*_*d*_ are the source and domain labels, respectively. Model blocks are represented as rectangles and losses as ellipses. The concrete part is a general framework of of adversarial domain matching. The dash lines represents the process of our uncertainty reweighting.

Previous methods, such as Tzeng et al. (2017), perform latent representation distribution matching in an adversarial manner. The intuition is to learn a domain invariant representation that only contains the label information. As in the concrete part of Figure 2, adversarial domain matching consists of three components: a source and target encoder Enc(·), a label predictor *C*(·), and a domain discriminator *D*(·).

The source encoder Enc(**x**_*s*_; ψ_*s*_) and label predictor *C*_ϕ_(·) are trained in a supervised-learning manner on $D_{s}$ by minimizing the cross-entropy loss as:


(1) $$\begin{array}{r} L_{c}=-E_{\left(x_{s},y_{s}\right)\sim D_{s}}\left[y_{s}^{⊤}\log\left\{C_{\phi }\left(\text{Enc}\left({\text{x}}_{s};\psi_{s}\right);\phi_{c}\right)\right\}\right], \end{array}$$

where *C*_ϕ_(·) is assumed to perform a softmax activation operation and $D_{s}$ is the joint distribution of the source. Once trained on the source dataset, both Enc(·;ψ_*s*_) and *C*_ϕ_(·) will be fixed during adaptation.

To minimize the impact of the discrepancy of features between source and target domains, the discriminator is learnt to classify the source and target feature domains, while the target encoder is trained to fool *D*, until *D* can no longer set an effective boundary between the source and target. During training, the discriminator is trained by minimizing the adversarial objective, $L_{\text{adv}}$,


(2) $$\begin{array}{r} \begin{array}{c} L_{\text{adv}}=-\;E_{{\text{x}}_{s}\sim X_{s}}\;\log D_{\theta }\left(\text{Enc}\left({\text{x}}_{s},\psi_{s}\right)\right)\\ -\;E_{{\text{x}}_{t}\sim X_{t}}\;\log\;1-D_{\theta }\left(\text{Enc}\left({\text{x}}_{t};\psi_{t}\right)\right)\;\; \end{array} \end{array}$$

And the target encoder is separately minimized by,


(3) $$\begin{array}{r} L_{\text{enc}}=-E_{{\text{x}}_{t}\sim X_{t}}\;\log\left(D_{\theta }\left(\text{Enc}\left({\text{x}}_{t};\psi_{t}\right)\right)\right), \end{array}$$

where we have inverted the labels relative to Equation (2) as in Goodfellow et al. (2014), which has the same properties of the original min-max loss used in GAN but results in stronger gradients for the target encoder.

### 2.2. Wasserstein Gradient Flows

Wasserstein uncertainty estimation is achieved via Wasserstein gradient flows (WGFs) (Villani, 2008). It is a generalization of gradient flows on Euclidean space. Formally, we first endow a Riemannian geometry (Carmo, 1992) on P(Ω). The geometry is characterized by the length between two elements (two distributions), defined by the second-order Wasserstein distance:


$$\begin{array}{l} W_{2}^{2}\left(\mu ,\nu \right)≜\underset{\gamma }{\inf}\left\{\int_{\Omega \times \Omega }\|\theta -\theta^{\prime }{\|}_{2}^{2}\text{d}\gamma \left(\theta ,\theta^{\prime }\right):\gamma \in \Gamma \left(\mu ,\nu \right)\right\}\;, \end{array}$$

where Γ(μ, ν) is the set of joint distributions over (**θ**, **θ**′) such that the two marginals equal μ and ν, respectively. The Wasserstein distance defines an optimal-transport problem, where one wants to transform μ to ν with minimum cost (Villani, 2008). Thus, the term $\|\theta -\theta^{\prime }{\|}_{2}^{2}$ represents the cost to transport **θ** in μ to **θ**′ in ν, and can be replaced by a general metric *c*(**θ**, **θ**′) in a metric space. If μ is absolutely continuous w.r.t. the Lebesgue measure, there is a unique optimal transport plan from μ to ν, i.e., a mapping *T*:ℝ^*d*^ → ℝ^*d*^ pushing μ onto ν satisfying *T*_#_μ = ν. Here *T*_#_μ denotes the pushforward measure (Villani, 2008) of μ. The Wasserstein distance thus can be equivalently reformulated as


(4) $$\begin{array}{r} W_{2}^{2}\left(\mu ,\nu \right)≜\underset{T}{\inf}\left\{\int_{\Omega }\|\theta -T\left(\theta \right){\|}_{2}^{2}\text{d}\mu \left(\theta \right)\right\}\;, \end{array}$$

Consider P(Ω) with a Riemannian geometry endowed by the second-order Wasserstein metric. Let {_μ_τ_}τ ∈ [0, 1]_ be an absolutely continuous curve in P(Ω) with distance between μ_τ_ and μ_τ+*h*_ measured by $W_{2}^{2}\left(\mu_{\tau },\mu_{\tau +h}\right)$. We overload the definition of *T* to denote the underlying transformation from μ_τ_ to μ_τ+*h*_ as **θ**_τ+*h*_ = *T*_*h*_(**θ**_τ_). Motivated by the Euclidean-space case, if we define ${\text{v}}_{\tau }\left(\theta \right)≜\underset{h\to 0}{\lim}\frac{T_{h}\left(\theta_{\tau }\right)-\theta_{\tau }}{h}$ as the *velocity of the particle*, a gradient flow can be defined on P(Ω) correspondingly in Lemma 1 (Ambrosio et al., 2005).

**Lemma 1**. *Let {_μ_τ_}τ ∈ [0, 1]_ be an absolutely-continuous curve in P(Ω) with finite second-order moments. Then for a.e. τ ∈ [0, 1], the above vector field **v**_τ_ defines a gradient flow on P(Ω) as ∂_τ_μ_τ_+∇_**θ**_·(**v**_τ_μ_τ_) = 0, where $\nabla_{\theta }\cdot \text{a}≜\nabla_{\theta }^{⊤}\text{a}$ for a vector **a***.

Function *F* above is lifted to be a functional in the space of probability measures, mapping a probability measure μ to a real value, i.e., F:P(Ω)→ℝ. *F* is the energy functional of a gradient flow on P(Ω). Consequently, it can be shown that **v**_τ_ in Lemma 1 has the form ${\text{v}}_{\tau }=-\nabla_{\text{x}}\frac{\delta F}{\delta \mu_{\tau }}\left(\mu_{\tau }\right)$ (Ambrosio et al., 2005), where $\frac{\delta F}{\delta \mu_{\tau }}$ is called the *first variation* of *F* at μ_τ_ (Doğan and Nochetto, 2022). Based on this, gradient flows on P(Ω) can be written in a form of partial differential equation (PDE) as


(5) $$\begin{array}{r} \partial_{\tau }\mu_{\tau }=-\nabla_{\theta }\cdot \left({\text{v}}_{\tau }\mu_{\tau }\right)=\nabla_{\theta }\cdot \left(\mu_{\tau }\nabla_{\theta }\left(\frac{\delta F}{\delta \mu_{\tau }}\left(\mu_{\tau }\right)\right)\right)\;. \end{array}$$

Intuitively, an energy functional *F* characterizes the landscape structure of the corresponding manifold, and the gradient flow (Equation 5) defines a solution path on this manifold. Usually, by choosing appropriate *F*, the landscape is convex, e.g., the Itó-diffusion case (Chen C. et al., 2018). This provides a theoretical guarantee of optimal convergence of a gradient flow.

## 3. Proposed Method

In domain matching, instances with high prediction uncertainty from the discriminator are usually less domain specific, thus can be easily transferred. Besides, samples with low domain uncertainty might contain too much domain specific information. Such samples may cause unreliable gradient and result in instability during adversarial training. Therefore, the discriminator prediction uncertainty can serve as a measure of cross-domain transferability between the source and target samples, enabling adaptive learning from easy to difficult instances.

### 3.1. Wasserstein Uncertainty Estimation for Probabilistic Discriminator

We consider learning posterior distributions for the parameters of the discriminator for the uncertainty estimation, instead of a point estimation. We further leverage the uncertainty of domain predictions to reweight source and target instances. The posterior distribution of complex models, e.g., the neural networks, is usually intractable. For computational convenience, traditional BNN learning typically assumes fully factorized Gaussian proposals as posterior approximation when adopting variational inference (Blundell et al., 2015; Hernández-Lobato and Adams, 2015). It is obvious that the factorized Gaussian posteriors usually lead to unreasonable approximation errors and underestimate model uncertainty (underestimate variances) (Liu and Wang, 2016). Further, particle-based variational inference methods (Chen C. et al., 2018), e.g., Wasserstein gradient flows, iteratively transports a set of particles to approximate the target posterior distribution, without making explicit assumptions about the form of the posterior and avoiding the aforementioned factorization assumption.

We consider a posterior distribution $p_{\theta }≜p\left(\theta |D\right)\propto p\left(D|\theta \right)p\left(\theta \right)$, where **θ** ∈ ℝ^*r*^ represents the parameter of domain discriminator. The canonical form is p(θ|D)=(1/Z)exp(Q(θ)).


(6) $$\begin{array}{r} \begin{array}{c} Q\left(\theta \right)≜\log p\left(D|\theta \right)+\log p\left(\theta \right)\\ =\overset{N}{\underset{i=1}{\sum }}\log p\left({\text{x}}_{i}|\theta \right)+\log p\left(\theta \right), \end{array} \end{array}$$

where potential energy is based on an i.i.d. assumption of the model, and *Z* is the normalizing constant, which is intractable if the discriminator model is a neural network. To apply WGFs for posterior approximation in domain discriminator, a variational (posterior) distribution for **θ**, denoted as μ(**θ**), is learned by solving an appropriate gradient-flow problem. To make the stationary distribution of the WGF consistent with the target posterior distribution, we define an energy functional characterizing the similarity between the current variational distribution and the true distribution *p*_**θ**_ as:


(7) $$\begin{array}{r} \begin{array}{c} F\left(\mu \right)≜-\underset{E_{1}}{\underset{︸}{\int Q\left(\theta \right)\mu \left(\theta \right)\text{d}\theta }}+\underset{E_{2}}{\underset{︸}{\int \mu \left(\theta \right)\log\mu \left(\theta \right)\text{d}\theta }}\\ =\text{KL}\left(\mu \|p_{\theta }\right)\;. \end{array} \end{array}$$

Note *E*_2_ is the energy functional of a pure Brownian motion (e.g., *U*(**θ**) = 0 in Equation 7). According to Equation (5), the first variation of functional *E*_1_ and *E*_2_ can be calculated as:


(8) $$\begin{array}{r} \frac{\delta E_{1}}{\delta \mu }=-Q,\;\frac{\delta E_{2}}{\delta \mu }=\log\mu +1\;. \end{array}$$

Substituting Equations (8) into (5) yields the specific PDE form of the WGF. The energy functional *F*(μ) defines a landscape determined by $D_{s}$, whose minimum is obtained at μ = *p*_**θ**_.

### 3.2. A Numerical Solution of WGFs

To solve the above WGF problem (Equation 5) we proposed to use particles, approximating μ with *M* particles ${\left\{\theta^{i}\right\}}_{i=1}^{M}$ as


(9) $$\begin{array}{r} \mu^{\left(h\right)}\approx \frac{1}{M}\overset{M}{\underset{i=1}{\sum }}\delta_{\theta^{i}}, \end{array}$$

where δ_**θ**_*k*__ is a delta function with a spike at **θ**_*k*_. Consequently, solving for the optimal μ is equivalent to updating the particles. We investigate the numerical solution to solve (Equation 5) via the discrete-gradient-flow method.

Discrete gradient flows (DGFs) approximate (Equation 5) by discretizing the continuous curve μ_*t*_ into a piece-wise linear curve, leading to an iterative optimization problem to solve the intermediate points denoted as ${\left\{\mu_{k}^{h}\right\}}_{k}$, where *k* denotes the discrete points, and *h* is referred to as the stepsize parameter. The iterative optimization problem is also known as the minimizing movement scheme (MMS) (Jordan et al., 1998), where for iteration *k*, $\mu_{k+1}^{\left(h\right)}$ is obtained by solving the following optimization problem:


(10) $$\begin{array}{r} \mu_{k+1}^{\left(h\right)}=\arg\underset{\mu }{\min}\;\text{KL}\left(\mu \|p_{\theta }\right)+\frac{W_{2}^{2}\left(\mu ,\mu_{k}^{\left(h\right)}\right)}{2h}\;. \end{array}$$

With particles approximating the μ in Equation (9), the evolution of distributions described by Equation (5) can be approximated with gradient descent on particles. According to Liu and Wang (2016), the gradient of the first term *F*_1_ ≜ KL(μ||*p*_**θ**_) can be easily approximated as:


(11) $$\begin{array}{r} \frac{\partial F_{1}}{\partial \theta_{k}^{i}}=\overset{M}{\underset{j=1}{\sum }}\left[-\kappa \left(\theta_{k}^{j},\theta_{k}^{i}\right)\nabla_{\theta_{k}^{i}}Q\left(\theta_{k}^{i}\right)+\nabla_{\theta_{k}^{j}}\kappa \left(\theta_{k}^{j},\theta_{k}^{i}\right)\right], \end{array}$$

where κ is the kernel function, which typically is the radial basis function (RBF) kernel defined as $\kappa \left(\theta ,\theta^{\prime }\right)=\exp\left(-\|\theta -\theta^{\prime }{\|}_{2}^{2}/h\right)$.

#### 3.2.1. Particle-Based Estimation of Wasserstein Distance

Unfortunately, the exact minimization of the Wasserstein distance $W_{2}^{2}\left(\cdot ,\cdot \right)$ over γ is in general computational intractable (Genevay et al., 2018; Salimans et al., 2018). Chen C. et al. (2018) uses a Sinkhorn-style algorithm to compute the Wasserstein distance but without iterative process assuming the parallel transport (Liu et al., 2019). It renders an inexact estimation with almost equal weights in γ. To overcome this issue, we consider an efficient iterative approach to approximate the Wasserstein distance based on the particle approximation. We propose to use the recently introduced Inexact Proximal point method for Optimal Transport (IPOT) (Xie et al., 2018) algorithm to compute the matrix T^*^. IPOT provides a solution to the original Wasserstein distance specified in Equation (4). Specifically, IPOT iteratively solves the following optimization problem using the proximal point method (Boyd and Vandenberghe, 2004):


$$\begin{array}{r} {\text{T}}^{\left(t+1\right)}={\arg\min}_{\text{T}\in \Pi \left(\text{x},\text{y}\right)}\left\{\langle \text{T},\text{C}\rangle +\beta \cdot B\left(\text{T},{\text{T}}^{\left(t\right)}\right)\right\}, \end{array}$$

where the proximity metric term $B\left(\text{T},{\text{T}}^{\left(t\right)}\right)$ penalizes solutions that are too distant from the latest approximation, and $\frac{1}{\beta }$ is understood as the generalized stepsize. This renders a tractable iterative scheme toward the exact Wasserstein distance. In this work, we employ the generalized KL Bregman divergence $B\left(\text{T},{\text{T}}^{\left(t\right)}\right)=\underset{i,j}{\sum }{\text{T}}_{ij}\log\frac{{\text{T}}_{ij}}{{\text{T}}_{ij}^{\left(t\right)}}-\underset{i,j}{\sum }{\text{T}}_{ij}+\underset{i,j}{\sum }{\text{T}}_{ij}^{\left(t\right)}$ as the proximity metric. Finally, we can update the particles in the *k*-th iteration with **T** computed based on IPOT and fixed when updating the particles:


$$\begin{array}{rc} \theta_{k+1}^{i}=\theta_{k}^{i}+ & \frac{h}{M}\overset{M}{\underset{j=1}{\sum }}\left[-\kappa \left(\theta_{k}^{j},\theta_{k}^{i}\right)\nabla_{\theta_{k}^{i}}U\left(\theta_{k}^{i}\right)+\nabla_{\theta_{k}^{j}}\kappa \left(\theta_{k}^{j},\theta_{k}^{i}\right)\right] \end{array}$$

(12) $$\begin{array}{rc} + & h\overset{M}{\underset{j=1}{\sum }}{\text{T}}_{ij}\left(\theta_{k}^{i}-\theta_{k-1}^{j}\right). \end{array}$$

### 3.3. Adversarial Domain Matching via Wasserstein Uncertainty Estimation

Given particles ${\left\{\theta_{k}\right\}}_{k=1}^{M}$, the prediction uncertainty of the domain discriminator can be estimated with different metrics. In our approach, we consider two uncertainty measures: entropy and variance uncertainty. Let *x* be a training sample, its domain prediction uncertainty can be estimated as,


(13) $$\begin{array}{r} U_{ent}\left(\text{x}\right)=H\left(\frac{1}{M}\overset{M}{\underset{k=1}{\sum }}D_{\theta_{k}}\left(\text{Enc}\left(\text{x};\psi \right)\right)\right) \end{array}$$

(14) $$\begin{array}{r} U_{var}\left(\text{x}\right)=\|\text{cov}\left({\left\{D_{\theta_{k}}\left(\text{Enc}\left(\text{x};\psi \right)\right)\right\}}_{k=1}^{M}\right){\|}_{2} \end{array}$$

where *H*(·) is the entropy function and cov(·) is the covariance operator. The norm of the covariance matrix corresponds to its largest eigenvalue, which is identical to the largest variance among all 1D projections of the particle outputs ${\left\{D_{\theta_{k}}\left(\text{Enc}\left(\text{x};\psi \right)\right)\right\}}_{k=1}^{M}$. Since (Equations 13, 14) are based in the particles solve from WGF, as in Section 3.2, *U*_*ent*_(**x**) and *U*_*var*_(**x**) are called *Wasserstein uncertainty estimation*. They describes the domain prediction uncertainty of the input samples with a domain discriminator.

For adversarial domain matching, we propose to reweight the source and target samples according to our domain prediction uncertainty. Given a training sample, its uncertainty weight can be evaluated as,


(15) $$\begin{array}{r} w\left(\text{x}\right)=\lambda \frac{U_{ent}\left(\text{x}\right)}{\log2}+\left(1-\lambda \right)\frac{U_{var}\left(\text{x}\right)}{U_{var}^{max}} \end{array}$$

where $U_{var}^{max}$ is the maximum variance uncertain in the current minibatch, and λ ∈ [0, 1] is a weighting parameter. Since the discriminator is a binary classifier, the largest entropy is bounded by log2. Hence we normalize the entropy-based uncertainty into [0, 1] by scaling it with log2.

We integrate our uncertainty weights into the adversarial domain matching in Section 2.1. For each particle *k* for the discriminator, *k* = 1, …, *M*, the adversarial objective can be modified as,


(16) $$\begin{array}{r} \begin{array}{c} L_{\text{ad}{\text{v}}_{\text{k}}}^{w}=-\;E_{x\sim p\left(X_{s}\right)}\;w\left(\text{x}\right)\log D_{\theta_{k}}\left(\text{Enc}\left(\text{x};\psi_{s}\right)\right)\\ -\;E_{x\sim p\left(X_{t}\right)}\;w\left(\text{x}\right)\log\left(1-D_{\theta_{k}}\left(\text{Enc}\left(\text{x};\psi_{t}\right)\right)\right). \end{array} \end{array}$$

The loss for the target encoder is modified as,


(17) $$\begin{array}{r} L_{\text{enc}}^{w}=-E_{x\sim p\left(X_{t}\right)}\;w\left(\text{x}\right)\log\left(\overset{M}{\underset{k=0}{\sum }}D_{\theta_{k}}\left(\text{Enc}\left(\text{x};\psi_{t}\right)\right)\right) \end{array}$$

The complete procedure for the proposed adversarial domain matching is illustrated in Algorithm 1. Such a matching mechanism can be implemented in virtually any domain adaptation algorithm based on adversarial domain matching. Specifically, in our experiments, we modify the adversarial training process of Wang et al. (2019) with our uncertainty reweighting. The results show that our method can yield remarkable improvements and effectively alleviate negative transfer in case of partial domain adaptation.

**Algorithm 1:** Adversarial domain matching with Wasserstein uncertainty reweighting.

Let ψ_*s*_, ψ_*t*_, be the parameters for source and target encoders. ${\left\{\theta_{k}\right\}}_{k=1}^{M}$ be the parameters for the samples discriminator particles.
**Input:**
Source and target data: {*X*_*s*_, *Y*_*s*_}, *X*_*t*_
Learning rates {γ_adv_, γ_enc_}
Batch size *B*
Number of particles *M*
Training source model, Enc(·;ψ_*s*_) and *C*(·), with *L*_c_
Fix Enc(·;ψ_*s*_) and *C*(·).
Initialize ${\left\{\theta_{k}\right\}}_{k=1}^{M}$
**while** not converge **do**
Draw random minibatch ${\left\{{\text{x}}_{s}^{i}\right\}}_{i=1}^{B}$, ${\left\{{\text{x}}_{t}^{i}\right\}}_{i=1}^{B}$
$\psi_{t}=\psi_{t}-\gamma_{\text{enc}}\nabla_{\psi_{t}}L_{\text{enc}}^{w}$
Calculate ${\left\{\nabla_{\theta_{k}}L_{\text{ad}{\text{v}}_{\text{k}}}^{w}\right\}}_{k=1}^{M}$
Update ${\left\{\theta_{k}\right\}}_{k=1}^{M}$ according to Equation (12)
**end while**

**Remark 2**. *The proposed method can be regarded as a scheme of adaptive importance sampling, which excludes the difficult instances at the earlier stage of adaptation and stabilize the adversarial training. Further, in partial domain adaptation, source classes not included in target will be predicted with very low domain uncertainty throughout training. These classes will be down weighted and ruled out during domain matching*.

## 4. Related Work

Unsupervised domain adaptation is based on the appropriate matching between the source and target distributions (Tzeng et al., 2014; Long et al., 2015, 2016; Sun and Saenko, 2016; Sun et al., 2016). Driven by the increasing popularity of the Generative Adversarial Networks (GANs) (Goodfellow et al., 2014), recent adaptation methods resort to matching the distributions in an adversarial manner. Long et al. (2015) and Tzeng et al. (2017) added a discriminator on the output of bottleneck layer in the model to distinguish features from different domains, while the feature encoders are trained to fool the discriminator so it cannot find an effective boundary that distinguishes between source and target instances. Zhang et al. (2018b) also add domain discriminators on the lower layers, which encourage domain specific information in shallower representations. Cao et al. (2017) and Cao et al. (2018) introduced the concept of partial domain adaptation, in which target classes are assumed to be a subset of the source. They reduce the effect of negative transfer by selecting out classes not present in the target according to the prediction frequency, however, their approaches are only moderate when the source and target label domains are the same. Cao et al. (2019) propose to identify samples from the redundant source classes through class-aware domain discrimination, however, they did not take into account the probabilistic domain uncertainty. In our approach, we propose to employ a probabilistic domain discriminator and reweight the source the target samples with the domain prediction uncertainty. Our uncertainty weights impose a meaningful curriculum for adversarial domain matching and can also select out samples from redundant source classes during partial domain adaptation.

## 5. Experimental Results

We denote our method as Wasserstein Uncertainty Domain Matching (WUDM). We evaluate WUDM on three domain adaptation benchmark datasets: the digits datasets, Office31 and Visda2017. In order to evaluate the effectiveness of our proposed Wasserstein uncertain estimation, we conduct an ablation test by using a domain discriminator with point estimation, denoted as UDM, where the uncertainty is estimated only with its prediction entropy as in Equation (13). UDM is an ablation study of our proposed Wasserstein Uncertainty Estimation, which is equivalent to estimating the domain uncertaintyusing the entropy uncertainty (Namdari and Li, 2019) with deterministic models. Source code will be released at https://github.com/RayWangWR?.

### 5.1. Datasets

#### 5.1.1. The Digits Datasets

We consider three digits datasets with varying difficulties: MNIST, SVHN and USPS, each containing 10 classes for digits 0-9. The encoder architecture for the digits images is the modified LeNet from Tzeng et al. (2017). For the domain classification, each sampled particle of the adversarial discriminator consists of 3 fully connected layers with 500 hidden units for the first two layers and 2 for the output. All images are converted to grayscale and rescaled to 28 × 28 pixels. Following the experiments of Tzeng et al. (2017), we consider three directions of transfer: SVHN → MNIST, USPS → MNIST and MNIST → USPS.

#### 5.1.2. VisDA2017

This is a dataset for the Visual Domain Adaptation Challenge from synthetic 2D renderings of 3D models to real images. It consists of 12 classes of objects shared by both domains, each with a very large number of instances. The architecture of the encoder for images in Visda2017 is a Resnet-50 (He et al., 2016) pre-trained on ImageNet. All the images are first resized to 256 × 256 pixels RGB images, then random cropped during training and central cropped during testing into 224 × 224 RGB images for the model input.

#### 5.1.3. Office31

This is a standard benchmark for domain adaptation widely used in computer vision, it consists of 4,652 images from 31 classes. These images are collected from three distinct domains: Amazon (A), Webcam (W) and DSLR (D). This is a relatively difficult dataset since the Webcam and DSLR contains very small amount of images, i.e., less than 10 for some classes, which may easily lead to overfitting during the adaptation process. In order to explore different combinations of large and small datasets for the source and target, we consider three transfer directions: A → D (large to small), W → A (small to large) and W → D (small to small).

The data pre-processing and experiment setting are the same as above for Visda2017 except that we use ResNet-50 with 31-dimensional output instead of 12. Due to the small size of Office31, we approach the task as fully transductive, where all labeled instances from the source and all unlabeled instances from the target are used during training and adaptation. This is the same for the experiments in Long et al. (2015), Ganin et al. (2016), and Tzeng et al. (2017). Complementary to Visda2017, Office31 will validate the performance of our method on small-scale datasets.

### 5.2. Balanced Domain Adaptation

#### 5.2.1. The Digit Datasets

We conduct experiments with all the 10 digits in the balanced setting. The results are shown in Table 1. Our method outperforms all the other baselines in all three directions, which demonstrates the effectiveness of our method in the standard balanced domain adaptation.

**Table 1:** Balanced domain adaptation on the digits datasets.

**Method**	**SVHN → MNIST**	**USPS → MNIST**	**MNIST → USPS**
LeNet LeCun et al., 1998	0.598	0.634	0.771
ADDA Tzeng et al., 2017	0.760	0.901	0.894
MCD Saito et al., 2018b	0.962	0.941	0.942
AdDropout Saito et al., 2018a	0.950	0.931	0.932
RAAN Chen Q. et al., 2018	0.892	0.921	0.890
JDDA-I Chen et al., 2019	0.931	0.970	-
EntroDA Wen et al., 2019	0.915	0.981	0.957
RUDA Wang et al., 2019	0.965	0.979	0.952
UDM	0.969	0.953	0.945
WUDM	**0.971**	**0.985**	**0.961**

*The values in bold means it is the highest in each column*.

Note that the proposed method outperforms simple model (point estimation) with a large margin. This indicated that our uncertainty weights with Equation (15) is more accurate in representing sample uncertainty, demonstrating the effectiveness of our Wasserstein uncertainty estimation.

### 5.3. Partial Domain Adaptation

#### 5.3.1. VisDA2017

Following Cao et al. (2018), we only reserve images of the first 6 classes of VisDA2017 in alphabetic order in the target domain (REAL-6, SYN-6), and all the images of the 12 classes are kept in the source domain (REAL-12, SYN-12). The results for SYN12 → REAL6 and REAL12 → SYN6 are shown in Table 2. Our method outperforms RUDA and the other baselines by a large margin. This validates the usefulness of our uncertainty reweighting for partial domain adaptation.

**Table 2:** Partial domain adaptation on VisDA2017.

**Method**	**Syn-12 → Real-6**	**Real-12 → Syn6**	**Average**
ResNet He et al., 2016	0.421	0.568	0.494
DANN Ganin et al., 2016	0.327	0.605	0.466
RTN Long et al., 2016	0.279	0.500	0.390
ADDA Tzeng et al., 2017	0.545	0.562	0.554
ADDA-mix Tzeng et al., 2017	0.543	0.605	0.574
PADA Cao et al., 2018	0.535	0.765	0.650
ENT Cao et al., 2019	0.706	0.708	0.707
RUDA Wang et al., 2019	0.700	0.846	0.773
UDM	**0.754**	0.711	0.733
WUDM	0.750	**0.864**	**0.807**

*The values in bold means it is the highest in each column*.

**Figure 3:**
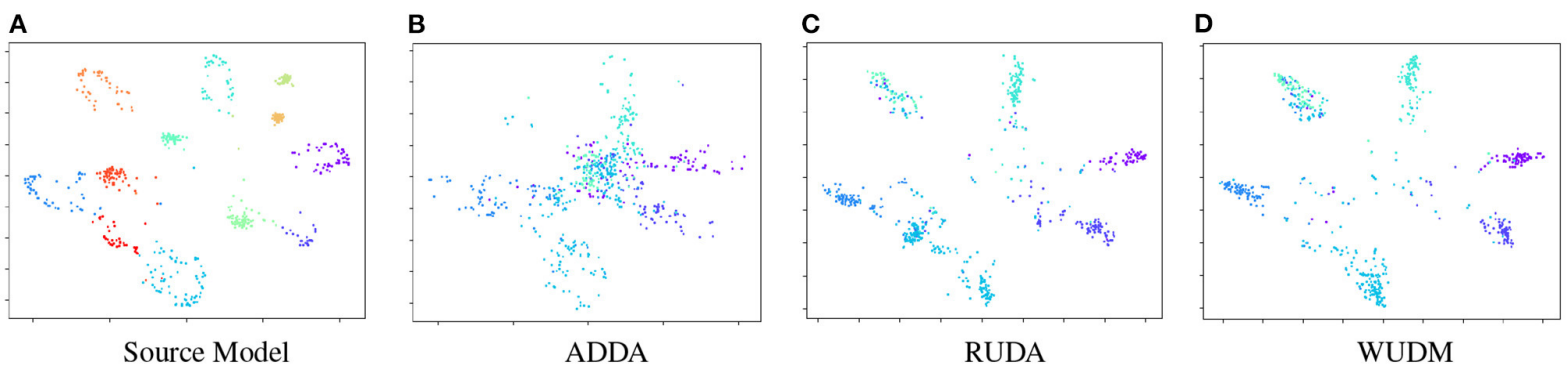
*t*-SNE embeddings of the representations for Syn12 → Real6. The features are embedded into the same subspace and visualized within the same scope. **(A)** is the source representation with 12 classes. **(B–D)** are the adapted target representation with 6 classes. The embedding from WUDM consists of 6 disentangled clusters, while others tend to be more entangled or contains excessive number of clusters.

In Figure 3, we visualize the source and target features of SYN12 → REAL6 in the same subspace with *t*-SNE. It can be shown that other methods tend to negatively transfer the target samples toward the redundant source classes. These samples will be misclassified by the source classifier and caused degraded performance for the adaptation. Our method promotes transfer within the same class, which preserves intra-class structure of target representation during domain adaptation. The resulting target representation is more discriminative and less entangled.

#### 5.3.2. Office31

We select the 10 classes shared by Office31 and Caltech-256 as our target labels. For each direction of adaptation, we use all the images of these 10 classes in the target split as the target domain (denoted as A10, W10, D10), and images from all the 31 classes in the source split as the source domain (denoted as A31, W31, D31). In Table 3, our method is better than RUDA and UDM in all three directions. These experiments validate the effectiveness of our uncertainty weighting in alleviating negative target transfer on small datasets as Office31.

**Table 3:** Partial domain adaptation on Office31.

**Method**	**A31 → D10**	**D31 → W10**	**D31 → A10**	**Average**
ResNet50 He et al., 2016	0.701	0.980	0.690	0.793
DANN Ganin et al., 2016	0.529	0.314	0.468	0.437
ADDA Tzeng et al., 2017	0.675	0.705	0.686	0.689
SAN Cao et al., 2017	0.813	0.986	0.806	0.868
IWAN Zhang et al., 2018a	0.790	0.990	0.895	0.892
PADA Cao et al., 2018	**0.865**	0.993	0.927	0.928
RUDA Wang et al., 2019	0.847	0.997	0.919	0.921
UDM	0.815	0.990	0.877	0.894
WUDM	0.860	**1.000**	**0.930**	**0.930**

*The values in bold means it is the highest in each column*.

Combined with the experiments of Visda2017, our method tend to produce larger performance improvement compared with the results from balanced domain adaptation. This is because partial domain adaptation suffers from larger degree of negative transfer, and our method can alleviate such effect by ruling out the irrelevant source classes and focusing on source samples that are more informative for target classification. The average accuracies of WUDM are higher then UDM in both Visda2017 and Office31. This validates the usefulness of our Wasserstein uncertainty estimation in the case of partial domain adaptation.

## 6. Conclusions

In this paper, we propose to reweight the source and target samples in domain adaptation with domain prediction uncertainty. For estimation of domain uncertainty, we employ a probabilistic domain discriminator and develop the Wasserstein uncertainty estimation, which can be easily integrated into concurrent adversarial domain matching. The resulting uncertainty weights impose an adaptive curriculum on domain adaptation that stabilize adversarial training and alleviate the effect of negative transfer in the case of partial domain adaptation. Experiments on several benchmarks show that our method achieves improved results on both balanced and partial domain adaptation.

## Data Availability Statement

Publicly available datasets were analyzed in this study. This data can be found here: http://yann.lecun.com/exdb/mnist/, https://paperswithcode.com/dataset/office-31, http://ai.bu.edu/visda-2017/.

## Author Contributions

All authors contributed to the article and approved the submitted version.

## Funding

It is funded by ECE, Duke University.

## Conflict of Interest

The authors declare that the research was conducted in the absence of any commercial or financial relationships that could be construed as a potential conflict of interest. The handling editor SX is currently organizing a Research Topic with the author(s) RZ.

## Publisher's Note

All claims expressed in this article are solely those of the authors and do not necessarily represent those of their affiliated organizations, or those of the publisher, the editors and the reviewers. Any product that may be evaluated in this article, or claim that may be made by its manufacturer, is not guaranteed or endorsed by the publisher.
