# Supplementary material for: Wasserstein Uncertainty Estimation for Adversarial Domain Matching
Source: Front Big Data. 2022 May 10;5:878716. doi: 10.3389/fdata.2022.878716 (PMC9128531; doi:10.3389/fdata.2022.878716)
Supplement: Supplementary file 1 [file Data_Sheet_1.PDF]

Supplementary Material

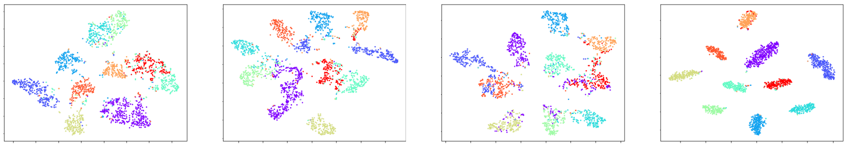

(a) ADDA (b) Ours (c) Ours (d) Ours

Figure 1: t-SNE plot of the feature domains for MNIST→USPS.

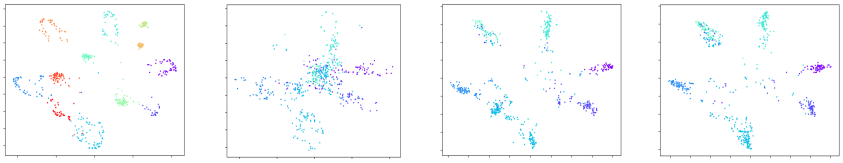

(a) Source Model (b) ADDA (c) RUDA (d) Ours

Figure 2: t-SNE embeddings of the representations for Syn12→Real6. The features are embedded into the same subspace and visualized within the same scope. (a) is the learnt source representation with 12 classes. (b)(c)(d) are the adapted target representation with 6 classes. The representation from our method consists of 6 disentangled clusters, while others tend to be more entangled or contains excessive number of clusters.
